# Micronodular pattern of organizing pneumonia: Case report and systematic literature review

**DOI:** 10.1097/MD.0000000000005788

**Published:** 2017-01-20

**Authors:** François Lebargy, Davy Picard, Jean Hagenburg, Olivier Toubas, Jeanne-Marie Perotin, Sebastian Sandu, Gaëtan Deslee, Sandra Dury

**Affiliations:** a Department of Respiratory Diseases, Reims University Hospital; b EA 4683 Medical and Pharmacological University of Reims; c Radiology Department; d INSERM UMRS 903; e Department of Cardio-Thoracic Surgery, Reims University Hospital, Reims, France.

**Keywords:** computed tomography, micronodules, organizing pneumonia

## Abstract

**Rationale:**

Organizing pneumonia (OP) is a clinicopathological entity characterized by granulation tissue plugs in the lumen of small airways, alveolar ducts, and alveoli. OP can be cryptogenic (primary) (COP) or secondary to various lung injuries.

**Patient concerns:**

We report the case of a 38-year-old male smoker with COP presenting in the form of diffuse micronodules on computed tomography (CT) scan and describe the clinical, radiological, and functional characteristics of micronodular pattern of organizing pneumonia (MNOP) based on a review of the literature including 14 cases.

Patients were younger (36.3 ± 15.5 years) than those with the classical form of OP. The clinical presentation was subacute in all cases with a mean duration of symptoms before admission of 14.5 ± 13.2 days. The radiological pattern was characterized by centrilobular nodules and “bud-in-tree” sign in 86.7% of patients. The diagnosis was based on histological examination of transbronchial (28.6%) or surgical biopsies (71.4%).

**Diagnosis:**

An associated condition was identified in 65% of cases and included illicit substance abuse (44.5%), myeloproliferative disease (33.5%), and infections (22%).

**Outcomes:**

Steroid therapy was effective in all patients with improvement of symptoms and documented radiologic resolution. No relapse was recorded.

**Lessons:**

MNOP should be recognized and distinguished from other diagnoses, mainly infectious bronchiolitis and disseminated tumor, as it requires early specific steroid therapy.

## 1 Introduction

Organizing pneumonia (OP) is a pathological entity characterized by the presence of polypoid granulation tissue in the lumen of bronchioles and alveolar ducts associated with variable degrees of interstitial and air space infiltration with mononuclear cells and foamy macrophages.^[1,2]^ Many conditions have been reported to be associated with OP, including pulmonary infection, connective tissue diseases, drug toxicity, and gastroesophageal reflux disease.^[3,4]^ In some cases, no etiology can be identified and OP is considered to be idiopathic, also called cryptogenic organizing pneumonia (COP).^[4]^ In most cases, the radiological presentation is characterized by multiple air space consolidations with a subpleural distribution or areas of ground glass lung infiltration.^[4–6]^ However, many other unusual presentations have also been reported.^[4,6]^

We report a case of acute OP presenting with a diffuse micronodular pattern mimicking miliary lung infiltration and discuss the clinical features of this unusual form of OP on the basis of a review of the literature.

## 2 Case report

**Figure 1:**
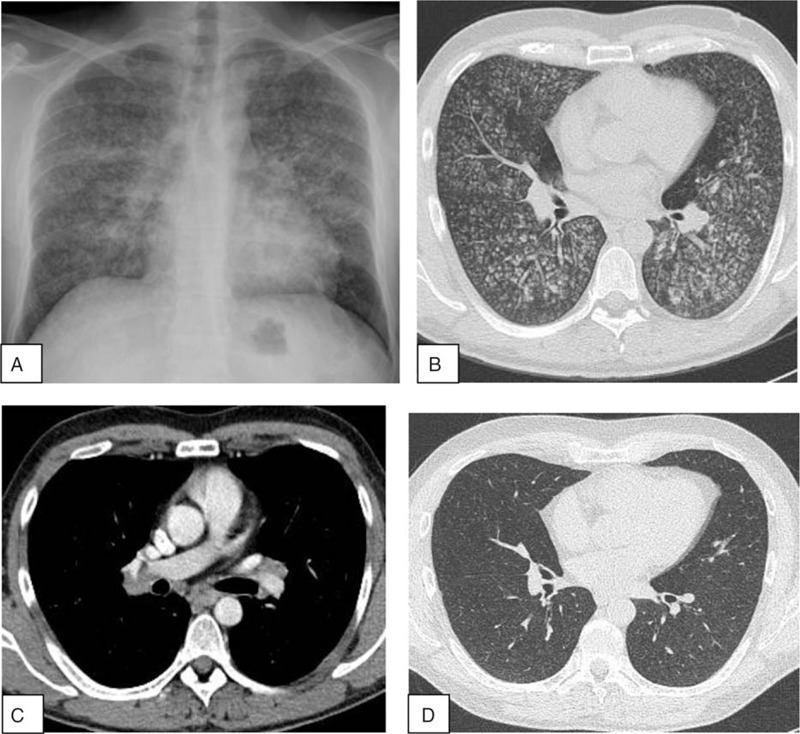
(A) Chest X-ray showing a micronodular pattern. (B) HRCT scan showing diffuse small centrilobular nodules. (C) Presence of interbronchial lymph nodes. (D) Improvement of miliary pattern after steroid therapy.

A 38-year-old man presented with an 8-day history of fever, myalgia, dry cough, dyspnea, and fatigue. He was a current smoker (10 cigarettes a day) and reported no occupational exposure. His medical history was unremarkable. Symptoms persisted despite antibiotic therapy with amoxicillin/clavulanic acid and roxithromycin. At admission, temperature was 38.5°C, respiratory rate was 24/minute, auscultation was normal and no extrathoracic signs were present. Chest X-ray showed diffuse micronodular opacities (Fig. 1A). High-resolution computed tomography (CT) demonstrated a profusion of well-delimited centrilobular micronodules, 2 mm in diameter, with subpleural sparing and a “bud in tree” pattern in some areas (Fig. 1B). Interbronchial lymph nodes were present (Fig. 1C). Arterial blood gases at 21% FiO_2_ showed PaO_2_ 66 mm Hg, PaCO_2_ 35 mm Hg, pH 7.44. Laboratory parameters were as follows: leukocyte count: 19.8 × 10^9^/L (85% polymorphonuclear cells), C-reactive protein: 305 mg/L and fibrinogen: 12 g/L. Serum electrolytes, and renal and liver function tests were normal, as well as serological tests for *Legionella pneumonia*, *Mycoplasma*, *Chlamydia*, *Coxiella psittaci*, hepatitis C and B viruses, and HIV. Antinuclear antibody, perinuclear antineutrophil cytoplasmic antibody (P-ANCA), cytoplasmic-ANCA, and cyclic citrullinated protein antibody tests were negative. Urine cocaine test was negative. Bronchoalveolar lavage (BAL) showed 400 × 10^3^ cells/mL, comprising 73% macrophages, 18% lymphocytes, 5% neutrophils, and 4% eosinophils. Golde score was normal (40). Testing for *Pneumocystis jiroveci*, *Mycobacterium tuberculosis*, and yeasts remained negative. Transbronchial biopsies were not contributive.

Pulmonary function test results were as follows: FEV_1_: 1.34 L (34%), FVC: 1.94 L (40%), FEV_1_/VC: 69%, TLC: 4.24 L (61%), DLCO: 11.59 mL min^−1^ mm Hg^−1^ (42%) and KCO: 3.83 mL min^−1^ mm Hg^−1^ (97%).

**Figure 2:**
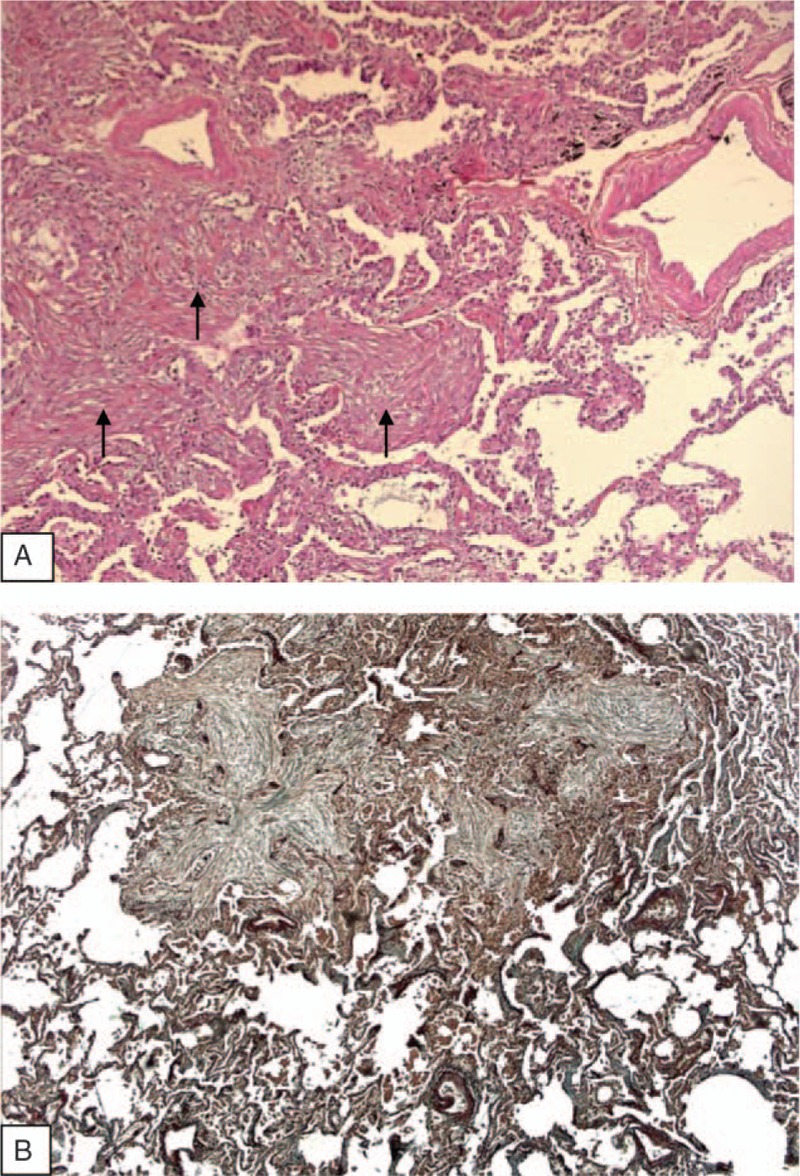
Histological examination of VATS lung biopsy. (A) Hematoxylin Phloxin Saffron staining (×2.5) proliferation of plump fibroblasts embedded in an edematous stroma forming intra luminal plugs in alveolar ducts and alveoli (arrows). Note the presence of a weak interstitial inflammatory infiltration at distance of fibrotic changes. No granuloma was observed in peribronchial areas or in interalveolar septa. (B) Orcein staining (×5). Buds of granulation tissue filling distal airspaces, extending from 1 alveolus to the next. Intraluminal fibrotic changes constitute the prominent lesion. No inflammation of vascular walls was observed.

To rule out an infectious disease, lung biopsies were performed under video-assisted thoracoscopy (VATS). Gross examination of slides disclosed a patchy nodular pattern. On microscopic examination, lung architecture was preserved. Nodules consisted in buds of granulation tissue fulfilling distal airspaces and extending from one alveolus to the next one, mimicking in some areas a butterfly pattern. Fibrotic buds disclosed bronchiolocentric predominance and in some areas extended into bronchiolar lumen. No granuloma was seen in lung parenchyma or in peribronchiolar areas. Mild inflammatory infiltrates was seen around fibrotic areas but interstitial inflammation was not present at distance of fibrotic plugs (Fig. 2). No pathogen was identified on Grocott and PAS staining and on biopsy cultures. This histological pattern was consistent with OP. The lack of marked interstitial inflammation and absence of granuloma ruled out other diagnoses such as sarcoidosis or hypersensitivity pneumonia.

Oral corticosteroid therapy was started at 1 mg/kg/day. Improvement of dyspnea was observed after 1 month of treatment, and CT micronodules had dramatically decreased (Fig. 1D). Steroids were tapered over 6 months and lung CT scan returned to normal with no signs of relapse at 5-year follow-up.

Informed consent was signed by the patient.

## 3 Methods

A PubMed database search including the terms “organizing pneumonia,” “cryptogenic,” “secondary,” “micronodules,” and “miliary,” identified 10 articles from January 1, 1997 (first description) to December 31, 2015, documenting 14 cases of OP presenting an exclusive micronodular pattern.^[7–17]^ Demographic, clinical, laboratory, imaging data, and associated conditions were analyzed from 15 case reports including our personal case.

Statistical analysis was performed using Epi Info statistical software (version 3.2.2). Data are expressed as mean ± standard deviation [minimum–maximum]. Comparisons were performed with Student *t* test and Fisher exact test. A *P*-value <0.05 was considered significant.

## 4 Results

The mean age at diagnosis was 37.6 ± 14.8 (Table 1). The M/F sex ratio was 9/6. Seven patients were smokers (58.3%). Acute or subacute presentation was observed in all cases with a mean duration of symptoms before admission of 14.5 ± 13.2 days. The most common symptoms were cough (93.3%), dyspnea on exertion or at rest (80%), sputum (40%), and mild fever in 46.7%, associated with a flu-like syndrome in 2 cases (data not shown). Crackles were present in 40% of cases. On CT scan, all patients presented a diffuse micronodular pattern with no apicobasal gradient. Micronodules were small (1–5 mm), with centrilobular distribution (86.7%) and demonstrated a “tree in bud” pattern in 7 cases. Interestingly, relative subpleural sparing was observed in some cases. Associated features included interbronchial lymph nodes (n = 2), ground glass pattern (n = 2), pleural effusion (n = 1), pneumomediastinum (n = 1). Diagnosis (n = 14) was obtained by surgery in 10 cases (VATS in 8 cases and open lung biopsy in 2 cases) or transbronchial biopsy by fiberoptic bronchoscopy in 4 cases. One patient declined surgical lung biopsy.^[16]^ Histological examination (n = 14) displayed a typical pattern of OP in 13 cases and acute fibrinous OP in 1 case. An associated condition was present in 9 cases, including myeloid proliferative disease (n = 3), infection (n = 2), marijuana exposure (n = 4). Four of these patients were severely immunocompromised. In the remaining 6 cases, no cause was identified and a diagnosis of COP was considered (Table 2). Although this study is limited by the small number of patients, no difference in terms of demographic, clinical, and imaging data was observed between COP and secondary OP.

**Table 1:**
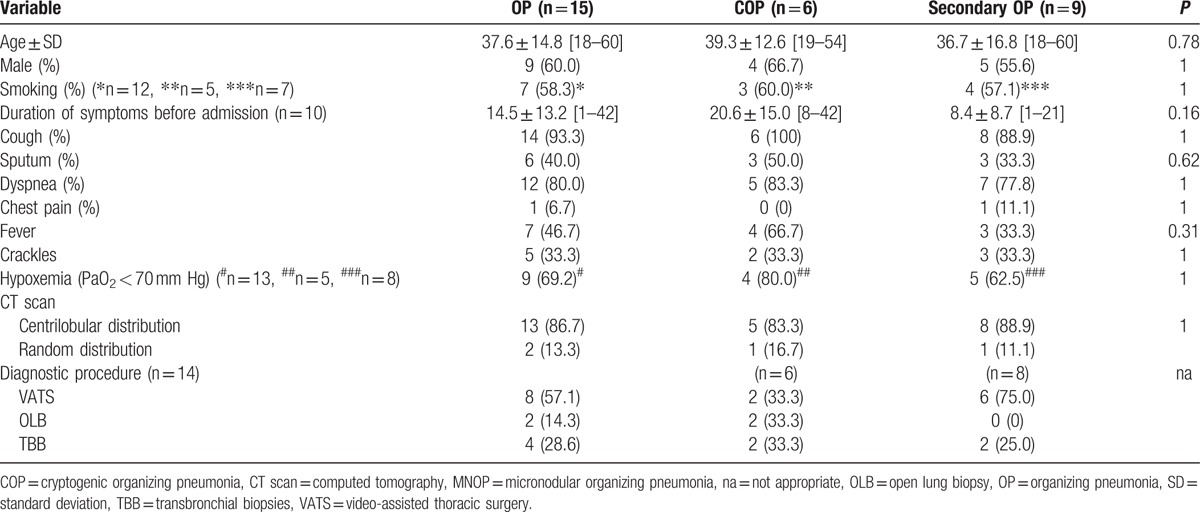
Clinical characteristics of patients with cryptogenic and secondary MNOP.

**Table 2:**
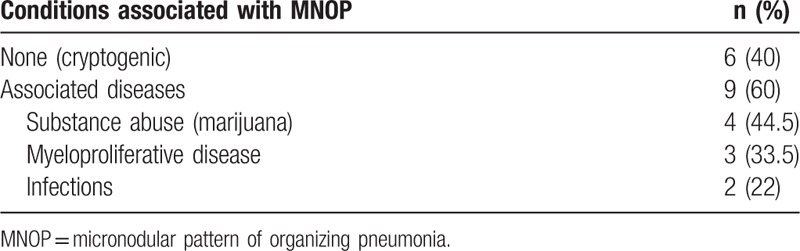
Conditions associated with MNOP.

Three patients died early due to hematologic malignancy (n = 2) or acute respiratory failure (n = 1). Improvement was obtained with steroid treatment in all remaining cases. Long-term follow-up was available for only 6 patients, ranging from 1 to 60 months. No recurrence was reported.

## 5 Discussion

Usual clinical and imaging presentations of OP have been well characterized in large series in the literature.^[5,6,18,19]^ The clinical course is usually subacute in 50- to 60-year-old patients.^[18]^ Cough, dyspnea, fever, and weigh loss are the main symptoms.^[18,19]^ Sparse crackles and bronchial breath sounds can be heard in areas of air space consolidation. Two main presentations have been described on CT scan, including patchy air space consolidation areas with a migratory course and ground glass opacities predominantly in the lung periphery.^[5,6,18,19]^ However, many other appearances have been reported, consisting of solitary or multiple nodules, focal pneumonia,^[20]^ perilobular consolidations, curved bands of consolidation, atoll sign.^[21–23]^ Our review focused on an unusual radiological presentation corresponding to diffuse micronodular pattern mimicking miliary lung infiltration.

The prevalence of micronodular pattern in OP (MNOP) in radiological series is estimated to range from 9.5% to 24% depending on whether OP is secondary or idiopathic and/or the subject's immune status.^[5,6,24]^ Patients are slightly younger than those with the classical form of OP.^[18,19]^ A male predominance is observed both in idiopathic and secondary MNOP, contrasting with the female predominance reported in 2 recent larger series of classical OP.^[25,26]^ The clinical presentation of MNOP does not differ from that of other forms of OP. Cough, dyspnea, and fever are the more prevalent symptoms. However, a rapidly progressive course appears to be more commonly observed in MNOP than in other forms.^[25–27]^ In this review, the duration of symptoms before admission was available in 10 patients. Five of these patients had a median duration of symptoms ≤8 days and the remaining 5 patients reported a duration of symptoms of 2 to 6 weeks. Nine patients presented hypoxemia and required oxygen supplementation, indicating poor clinical tolerance. A similar CT scan pattern was observed in all the cases with widespread lung micronodules without diffuse ground glass opacities, airspace consolidation, or cavitation. Interestingly, micronodules did not show random but a centrilobular distribution with “bud in tree” pattern in most cases, suggesting a primarily bronchiolar disorder such as infectious bronchiolitis or hypersensitivity pneumonia.^[28]^ Nevertheless, disseminated tuberculosis was considered as differential diagnosis in 5 cases and led to initiation of antituberculosis therapy. In the remaining cases, empirical antibiotic therapy was administered for suspected community-acquired bronchiolitis. In all but one of the published cases, invasive diagnostic procedures were performed due to the inefficacy of antiinfectious therapy. Video-assisted thoracoscopic or open lung biopsy was considered to provide the best tissue specimens to establish the histological diagnosis of idiopathic interstitial pneumonia.^[29]^ However, in large series of OP, the histological diagnosis was obtained on transbronchial biopsy specimens from 31% to 67% of cases.^[25–27]^ In the present review, the diagnosis was established on TBB specimens in 28.6% of patients. However, the sensitivity of the TBB procedure to demonstrate this micronodular pattern could not be assessed as TBB was performed in only 5 patients of this review (data not shown).

As previously reported, OP may be cryptogenic (COP) or secondary to various conditions, including drugs and substance abuse, infections, connective tissue disorders, solid tumors, and hematologic malignancies.^[3,4,25,27]^ In large series, secondary OP represented 24% to 45% of all cases of OP.^[25,27]^ Interestingly, MNOP was associated with another disease in 65% of cases (Table 2), mainly illicit substance abuse and myeloproliferative disease. The nodular pattern of OP has been known for a long time to be more frequently observed in immunocompromised patients. In the present review, MNOP was associated with hematologic malignancy^[11,12,14]^ or renal transplantation^[13]^ in 4 patients, corresponding to 29% of the overall cohort, but 44% of all cases of secondary OP. The results of this review are in agreement with those of a series of 43 consecutive patients with biopsy-proven OP, showing that nodules on CT scan were present in 55% of immunocompromised but in only 22% of immunocompetent patients.^[6]^

As in other forms of OP, steroid therapy is markedly effective with rapid improvement of symptoms and radiological resolution in most cases. In classical forms of OP, relapses occur in 58% of cases, but do not adversely impact outcome.^[26]^ No relapse was reported in the present review. Of note, 1 marijuana smoker died from acute respiratory failure.^[16]^

In conclusion, this literature review highlights the clinical and radiological characteristics of an unusual micronodular pattern of cryptogenic or secondary OP. Subacute presentation and radiological features of widespread bronchiolar nodules should lead to consider differential diagnoses such as infectious bronchiolitis especially in immunocompromised patients, hypersensitivity pneumonia or disseminated tumor. The definitive diagnosis requires transbronchial biopsy or video-assisted thoracoscopic lung biopsy. Steroid therapy usually allows improvement of symptoms and resolution of radiological signs.
